# Evaluating the Representation and Responsiveness of the United Nations Commission on the Status of Women (CSW) to Diverse Women Populations Worldwide

**DOI:** 10.3389/fsoc.2019.00041

**Published:** 2019-07-09

**Authors:** Meg Rincker, Marisa Henderson, Renato Vidigal, Daniel Delgado

**Affiliations:** ^1^Department of Political Science, Economics, and World Languages and Cultures, Purdue University Northwest, Hammond, IN, United States; ^2^European Institute, Columbia University, New York, NY, United States; ^3^Department of Economics, American University of Beirut, Beirut, Lebanon; ^4^Edmund A. Walsh School of Foreign Service, Georgetown University, Washington, DC, United States

**Keywords:** non-governmental organization, United Nations, women's empowerment and gender equality, democratization, intersectionality, policy priority, GIS, mixed-method

## Abstract

The United Nations Commission on the Status of Women (CSW) is “the principal global intergovernmental body exclusively dedicated to the promotion of gender equality and the empowerment of women.” The continued success of the CSW's mission is critical. Under the guise of needing competent male leaders to protect them, women and girls in many countries nonetheless suffer dire consequences from international conflict and war. While females may send mostly male loved ones into armed conflict, women and girls themselves face economic sanctions, poverty, displacement, and heightened risk of sexual violence, in an environment that already fails to respect their equal human rights with men. It is no accident that in most countries, females are de facto excluded from positions of power where they could try to prevent such conflict (Enloe, 2014), and the CSW is actively working to overcome this reality. In this article, we use a mixed-method approach to evaluate the level of United Nations Commission on the Status of Women (CSW) representation and responsiveness to diverse women populations as reflected in (a) state delegations' participation in formal session, (b) NGO participation, and (c) NGO priorities. We find that the CSW displays a high level of representation and responsiveness to diverse state delegations across UN regions in terms of their speaking time in formal session. The CSW is at best moderately representative of women's NGOs through regional advance consultations, and the level of responsiveness to women's NGOs is low. This is due to: costly barriers to entry to gain ECOSOC status, large underrepresentation of some UN Regions, and the fact that during formal session, women's NGOs participate primarily at parallel off-site events. Although the CSW passed important resolutions, its representation and responsiveness to women's NGO priorities is lower than it should be. We base our evaluation by comparing our surveys of attending and non-attending women's NGOs that showed top priorities that the 2010 CSW did not discuss or pass resolutions on such as: land rights, sex trafficking, internet access for women, and the effects of climate change on women. We contend that during 1970–1994, when World Conferences on Women occurred, parallel transnational advance consultations among women's NGOs in tandem with CSW regional consultations created a broadening of the CSW's agenda. The CSW rationally anticipated different kinds of feminism beyond liberal feminism and a broader set of priority issues for diverse women populations. We therefore argue for a Fifth World Conference on Women to increase the representativeness and responsiveness of the CSW.

## Introduction

The United Nations Commission on the Status of Women (CSW) is “the principal global intergovernmental body exclusively dedicated to the promotion of gender equality and the empowerment of women” (UN Women, a)^1^. Beginning in June 21, 1946 when an Economic and Social Council (ECOSOC) resolution elevated it from a sub-commission of the UN Commission on Human Rights to a commission, the CSW has been the site of landmark achievements in global women's rights. The CSW's achievements include contributing gender-inclusive language in the 1948 *Universal Declaration of Human Rights*, collecting global comparative data on the rights and status of women worldwide, and passing two declarations and three major binding multi-country conventions (including the 1979 *Convention on the Elimination of all forms of Discrimination Against Women*, CEDAW)^2^. The CSW founded International Women's Year and the UN Decade for Women. The CSW is also responsible for setting in motion three World Conferences on Women, monitoring the Nairobi Forward-looking Strategies, mainstreaming gender issues into UN development conferences, working on the implementation of the Beijing Declaration and Platform for Action (BDPfA), and developing the outcome of the twenty-third special session of the UN General Assembly (United Nations, b)^3^ Over the course of its history, the CSW has played a key role in getting gender equality mainstreamed included in the UN's policy agenda as a Millennium Development Goal and a Sustainable Development Goal. The CSW has also engendered many UN programs: increasing female rights to hold property, developing programs to end violence against women and girls, and increasing female access to education, health, and self-determination.

The CSW has achieved all of these landmark results in a United Nations (UN) framework, in which the CSW faces important external and internal obstacles. Externally, the UN recognizes state sovereignty and therefore requires states to cooperate on shaping human rights and women's rights policies and voluntarily implement them. The CSW as part of the UN then navigates in difficult waters externally. At worst, state leaders' views on women's empowerment can range from open opposition to women's rights and therefore appointment of CSW delegates who oppose progress, to sanguine support and failure to provide adequate resources, to circumvention of the CSW to demonstrate superiority rather than the end goal of advancing women's rights. Even leaders that agree and work with the CSW's process can cave to pressure from citizens who see CSW policies as infringement on a country's sovereignty (see Baldez, 2014). Internally, the CSW faces similar obstacles, as CSW delegations and women's NGOs may face leaders who oppose the type of feminism they espouse, divesting needed resources to participate and creating a chilly climate for those outside the norm. Even cordial disagreement on pathways to women's empowerment can make it difficult to reach consensus on the best means or language for a resolution to pass the CSW under time constraints. All this being said, the CSW's history of remarkable accomplishments in the face of adversity mean that the institution must remain strong to advance women's empowerment in challenging times. In this article, we use a mixed-method approach to evaluate the level of United Nations Commission on the Status of Women representation and responsiveness to diverse women populations as reflected in (a) state delegations' participation in formal session, (b) Non-Governmental Organization (NGO) participation, and (c) NGO priorities.

After describing the CSW's role in gender equality policy, we examine the representativeness and responsiveness of the CSW to diverse women populations. We employ the methodological strategy of triangulation (Tarrow, 1995), combining quantitative and qualitative analysis to create a more comprehensive and nuanced evaluation. First, we use quantitative analysis to study speaking time in formal session, and find that the CSW displays a high level of representation and responsiveness to diverse state delegations regardless of their level of economic development. Second, we examine the CSW's representation and responsiveness to women's NGOs through advance regional consultations before the formal session to discuss the current priority theme issue as it applies to women of that region of the world. We use institutional analysis, examining the CSW/ ECOSOC rules for women's NGOs to gain necessary consultative status to be able to participate directly in the 2010 CSW meetings. Our analysis suggests that onerous rules and costs may limit CSW responsiveness to diverse women populations. We empirically test this proposition, using Geographic Information Systems (GIS) to map all internationally visible women's NGOs in 2010 and compare these with a map of those that participated in 2010 CSW meetings. Our analysis shows regional overrepresentation of the United States, and underrepresentation from the UN Regions of Latin America, Africa (particularly the Middle East within it), and Eastern Europe (particularly Russia). To address critics who suggest that advance consultation is the main pathway by which the CSW responds to diverse women populations, we conduct online semi-structured survey interviews of women's NGOs (attending and non-attending) about their policy priorities, and compare their priorities to the resolutions passed during the 2010 CSW meetings.

A key contribution of this research is that we apply to the CSW at what Bachrach and Baratz (1962) describe as the two faces of power. We examine the viewpoints of both women's NGOs who attended the 2010 CSW meetings, and those that did not attend, about their top policy priorities. This research design helps us to see not just whether the CSW agenda matches participating NGO priorities, but also what is not on the CSW's agenda but should be. While our survey size is small, we demonstrate that our surveys are reflective of the geographical distribution of women's NGOs by the five UN regions. We find that although the CSW passed resolutions in important areas in its 2010 formal session, the CSW did not address some top priority issues emerging across regions. Triangulating our findings with historical, theoretical and empirical research on the CSW, we show that when more women's NGOs participate in the policymaking process through the four World Conferences on Women, the CSW's agenda rationally anticipates and broadens to include the concerns of other feminisms like women and poverty, and women in conflict. We find that while the CSW is highly representative and responsive to diverse women populations via official state delegation speaking time in formal session, the CSW is moderate at best in terms of including diverse women's NGOs before or during its formal meetings, or responding to the top policy priorities of women's NGOs worldwide. We conclude that a Fifth World Conference on Women is consistent with transnational women's NGO collaboration feeding into the CSW's regional consultation process, allowing the views of diverse women's NGOs inserted into the UN gender equality policymaking agenda (Fifth World Conference, 2017; Goetz, 2018).

Since its inception, the CSW has been comprised of members who represent their governments in an official capacity, reflecting the interests of their governments, as well as women's NGOs. The CSW is comprised of one state representative from each of an elected 45 member states of the United Nations. These 45 states are “elected by the Economic and Social Council on the basis of equitable geographical distribution across the UNs five regional groups: 13 members from Africa, 11 from Asia, nine from Latin American and Caribbean, eight from Western Europe and other States, and four from Eastern Europe, for a period of 4 years^4^”. Because the key actors in the CSW are state delegations, we first will examine the points of view representation and responsiveness to diverse women populations as reflected in state delegations' participation in debates. The Bureau of the CSW organizes preparations for the annual CSW Sessions, and serves for 2 years. Beginning in 2002, the CSW held an opening session for the subsequent CSW right after the closing of the previous CSW session, to elect the chairperson and Bureau members, one from each of the five UN regions. The CSW's methods of work consist of preparatory and consultative meetings over the year, culminating in a two week session in New York in March.

The CSW is comprised of state-appointed actors that seek to advance state interests on women's empowerment and gender equality, so some may question why CSW members meeting formally for just two weeks with little compensation would respond to women's NGOs around the world. We advance two main reasons why the CSW should represent and respond to women's NGOs. First, research shows that feminist mobilization in civil society i.e., women's organizations are the crucial factor explaining cross-national variation in policy development (Htun and Weldon, 2012). Second, the CSW's founding documents all acknowledge the information asymmetry that NGOs possess about the situation of diverse women on the ground in various countries, and recognize the role that women's NGOs play in aligning UN policies to what women need^3^.

Because women's NGOs are often critical of the CSW, over time, the CSW has modified women's NGO “modalities of work,” allowing only the participation of ECOSOC-approved groups, and moving them offsite to the NGO Forum (Owen, 2016). To be fully representative, the CSW would implement proposals from a diversity of women's organizations to dismantle barriers based on gender, race, class, and postcolonial status to empower women. The UN's processes have heard and been responsive to different women's groups and feminisms, but acknowledged them only in rhetoric, while in action mainly upholding liberal feminism in devising policies focused on reforming states, and removing discriminatory laws (Arat, 2015).

The CSW engages in regionally based consultation with women's groups particularly on priority theme issues that constitute an important avenue for representation and responsiveness to diverse women's populations. However, we argue that CSW policymaking is at its best when it anticipates prolonged and sustained transnational women's NGO involvement, such as in the lead up to the World Conferences on Women (WCW). In the lead up to WCW, women's NGOs meet within and across regions to strategize on how to address women's equality across regions, not just within regions.

How should we evaluate whether the CSW represents the claims of diverse women to empower women and bring about gender equality? Women's interests are plural, diverse, and sometimes conflicting. Women's NGOs appeal to the needs of women by making claims on behalf of women (Saward, 2010) to political elites in states and international organizations. Arat argues that in rhetorical terms, the UN has represented a broad diversity of women's claims and diverse feminisms described more below. However, Arat argues that when the UN identifies measures of women's empowerment they often reflect liberal feminism. She argues “the overall women's rights approach of the UN is still informed by the demands and expectations of liberal feminism” (2015, p. 674). Liberal feminism

demand[s] equal opportunities for women's education, electoral rights, economic participation, equal access to the public domain, and integration into all male institutions, liberal feminism seeks gradual change through legislative reform and anti-discrimination laws; it considers the state an apparatus that can be used to create equal opportunities for women and to establish gender equality (qtd. in Arat, 2015, p. 676).

Arat argues that when the UN chooses an indicator of women's empowerment reflecting liberal feminism, such as the percentage of women in parliament, this indicator serves to strengthen certain countries that then appear empowering and successful, even though they may only be benefiting a few women at the top of the socio-economic strata. Alternatively other countries on this indicator may appear unsuccessful on women's empowerment, even while they may be taking very impactful steps to expand women's empowerment on a broad level (such as maternal mortality and reproductive rights).

Political actors motivated by other feminisms like Marxist feminism and post-colonial feminism have challenged the UN. These approaches cite different causes of and solutions to women's subordination, and have at times been the subject of UN action, when the relative power of states has changed in the UN. Arat describes how, during the 1960s, Cold War dynamics sharply defined the UN. The Soviets sought to control UN discourse over women's empowerment through Marxist feminist ideas. “Marxist [feminism] sees the emancipation of women as possible only through the emancipation of the working class by a proletarian revolution that would eliminate private ownership of the means of production, bring women to the production process as the equals of men, and treat child rearing as a social/collective responsibility” (Arat, 2015, p. 676). After decolonization, during the 1970s and 1980s, many countries broke away from former empires, and formed the non-aligned movement, using their new collective voting power in the UN to challenge the United States and western powers. Post-colonial feminism emerged; identifying that gender, class, racial oppression, and western feminism are causes of the economic dependence of many countries, and the oppression of women. The BDPfA included third world feminists' concerns, stressing “the problems of poverty, economic inequalities, and militarism; it openly criticizes the negative impacts of structural adjustment policies and affirms the need to address the “structural causes of poverty,” (Arat, 2015, p. 679) as well as a need for transformational change.

We apply Arat's insights, examining the processes of the CSW itself, including identifying which women's NGOs worldwide participate in CSW events, and how successful they are at influencing CSW policy-making in setting the agenda or pushing for passage of CSW resolutions in areas they deem as top priorities. As policy entrepreneurs, women's NGO leaders discuss their priorities in places like directories, websites, newsletters, protests, and speeches.

The participation of women's NGOs across UN regions in accordance with the CSW's own distribution is crucial to CSW representativeness and responsiveness. Htun and Weldon (2012) argue that there are three reasons why “women's autonomous organizing has played such a critical role” in feminist policymaking. “First, women organizing as women generate social knowledge about women's position as a group in society…with “an oppositional consciousness as well as a set of priorities that reflect their distinctive experiences and concerns as a group.” Second, the issues women's organizations bring up like violence against women “challenge, rather than work within established gender roles in most places” meaning that insider democrats are more reluctant to risk being fired for bringing up issues that require major societal changes. The third reason is agenda-setting: “[w]hen women's movements are organized autonomously they do not need to justify within a larger organization like a political party, why their issue matters if it is important “only” to women (Htun and Weldon, 2012, p. 533). In sum, women's NGOs have special knowledge about, independent space to raise, and a natural sense of urgency about issues affecting women that together help them identify gaps or shortcomings in women's empowerment initiatives. Adequate representation in the CSW by women's NGOs from different UN regions can allow for articulation of diverse feminisms beyond liberal feminism.

The CSW's mission statement, the ECOSOC resolution creating the CSW, the BDPfA, and other resolutions, all acknowledge the information asymmetry that women's NGOs possess about the situation of diverse women on the ground in various countries. The CSW styles its own mission as representing and responding to civil society because the CSW invites civil society organizations to participate in advance consultations and the session itself:

*During the Commission's annual two-week session, representatives of UN Member States, civil society organizations and UN entities gather at UN headquarters in New York. They discuss progress and gaps in the implementation of the 1995 Beijing Declaration and Platform for Action, the key global policy document on gender equality, and the 23rd special session of the General Assembly held in 2000 (Beijing*+*5), as well as emerging issues that affect gender equality and the empowerment of women*^1^.

By including them in advance consultations and the CSW session, the CSW implies that women's NGOs are an important reality check on state reports on “progress and gaps” in implementing the BDPfA, as well as on newly emerging issues that states may not yet know about. Molyneux and Razavi (2005, p. 983) lament that while:

*[t]he 1995 Fourth World Conference on Women (the ‘Beijing Conference') was a landmark in policy terms, setting a global policy framework to advance gender equality. Ten years after Beijing, in March 2005, the UN's Commission on the Status of Women presided over an intergovernmental meeting in New York to review the progress achieved on the commitments made in the Beijing Declaration and Platform for Action. This “Plus 10” event was decidedly low key*.

Molyneux and Razavi discuss that there has been progress in many countries on issues like education and economic growth. However, there is much variation in gender equality across countries and policy areas, and progress is not always linear. “Human rights and women's agendas and the entire multilateral framework within which the gains of the 1990s were made have been weakened by the current global political crisis occasioned by terrorism, militarism, the war on Iraq and hostility to unilateralism” (Molyneux and Razavi, 2005, pp. 1004–1005). During times when public or elite support for women's empowerment is low, women's NGOs have and continue to play an important role in the CSWs agenda-setting, and implementation. The CSW website says: “[t]he active participation of non-governmental organizations (NGOs) is a critical element in the work of the Commission…NGOs have been influential in shaping the current global policy framework on women's empowerment and gender equality: the [BDPfA]^5^.” Yet the CSW does not adequately institutionalize consultations with women's NGOs, who possess first-hand knowledge about what women need, and who are more critical of their state's progress on than state-appointed CSW representatives. For example, numerous women's NGOs signed a Final Statement before the 2010 CSW meetings, noting:

*The Declaration on the occasion of the fifteenth anniversary of the Fourth World Conference on Women [was] agreed ahead of time and adopted without consultations with civil society…[it] appears to overstate the progress made, and to ignore the slow and partial nature of implementation. It underestimates the degree and types of challenges that remain for women in their multiple identities, including the persistence of all forms of violence against women (“Final Statement*, *2010*”*, p. 1)*.

This article builds on Arat's (2015) work to evaluate the level of the CSW's representation and responsiveness to different women populations as reflected in (a) state delegations' participation in debates, (b) NGO participation, and (c) NGO priorities.

## Materials and Methods

We focus our data collection on the 2010 CSW meetings, held 15 years after the Beijing Conference. We employ Tarrow's strategy of triangulation. As Tarrow discusses, “[t]riangulation is particularly appropriate in cases in which quantitative data are partial and qualitative investigation is obstructed by political conditions” (Tarrow, 1995, p. 473). Because evaluation of the representativeness and responsiveness of the CSW to diverse women's organizations is not merely something we can ask CSW members about to get a full evaluation, we use a combination of quantitative and qualitative indicators together to create a more comprehensive and nuanced picture. We employ quantitative analysis of state delegation speaking time in CSW formal session, GIS analysis of which women's NGOs attend the CSW, and semi-structured surveys of attending and non-attending women's NGOs about their top policy priorities. We triangulate these data with institutional analysis of ECOSOC rules for NGO participation, historical analysis of NGO participation in the CSW, theoretical work on the ideological breadth of the CSW's agenda over time, showing that there is underrepresentation of most regions since Beijing. When we combine this reality with policy analysis of the resolutions passed by the 2010 CSW, our conclusion and policy recommendation is clear: we need a Fifth World Conference on Women. We discuss each step in turn.

First, we examine the CSW's representation and responsiveness to diverse women populations by examining speaking time of state appointed representatives sitting in the formal 2010 CSW session. We do this because the CSW is at its core an international body where state-appointed representatives convene and deliberate on how to advance the status of women and girls. One way to get a sense of the substance of the official CSW meetings and the power dynamics involved is to examine speaking time at the session by country, by P-5 status and by leadership roles. Analysis of speaking time in a deliberative body by identity characteristics such as gender, race, sexual orientation, social class, and other factors is a common method of examining the political power of differently positioned participants (Kathlene, 1994, 1995). We argue that examining speaking time by state appointed delegates to the CSW is a visible outward sign of what the CSW does and lays the groundwork for further analysis of the CSW's multi-faceted responsiveness to diverse women populations. We acknowledge that more speaking time is not equivalent to more power; it is possible that certain state delegates could speak infrequently in formal session but yet affect the priorities or draft documents prepared prior to session. This is why we subsequently examine other pathways by which diverse women's populations to receive representation and responsiveness by the CSW.

Second, we examine the representation and responsiveness of the CSW to diverse women's populations through the consultative work that the CSW did with women's NGOs in the extensive deliberations and lead-up to the 2010 CSW Formal Session. As part of the CSW's Programme of Work, in the lead up to the Formal Session, the CSW engages in regional consultations with women's NGOs on the priority theme issue. The priority theme for 2010 was the 15-year Review of the implementation of the Beijing Declaration and Platform for Action, the outcomes of the twenty-third special session of the General Assembly and its contribution to shaping a gender perspective toward the full realization of the Millennium Development Goals (UN Women, a)^1^. While we applaud the work of the CSW in preparing regional-specific reports and soliciting feedback from diverse NGOs on these reports, we note a few concerns. We discuss how the CSW sets the priority theme for regional consultation, rather than emerging bottom-up from diverse women populations within each region. We argue that the CSW may be prioritizing discussion and attention on an issue area that is not a top priority for women within a region. Even if the priority theme is broad enough to allow diverse women's populations within a region to advance their prime concerns and strategies, it is also up to minimally resourced CSW leaders to draw best practices or identify cross-regional trends, after conducting regional consultations.

Third, building on our analysis of state-representative speaking time, and CSW advance regional consultations with women's NGOs, we examine whether women's NGOs from diverse regions participate in the CSW, and whether the CSW responded to their policy priorities in the form of resolutions passed during the 2010 CSW session. Specifically, we describe ECOSOC requirements for a women's NGO to apply for consultative status and discuss how these rules themselves might limit the diversity of women's NGOs that can attend, and either impact each other or the CSW. We empirically evaluate the diversity of women's NGOs that did participate in the 2010 CSW session against those that were internationally visible in 2010, showing important gaps in participation from some regions.

To do this, we use Geographic Information Systems (GIS) analysis to map all internationally visible women's NGOs in 2010 from the 2012 *International Directory of Women's Organizations* (IDWO-2010), when there were 1,760 such groups. The methodology section of this Directory notes that it is the most comprehensive and up-to-date of its kind worldwide, and that:

*a wide range of women's organization activity are covered from art to culture; business to education; gender equality to human rights; health to reproduction; families to development; politics to global leadership; women's empowerment; civil society promotion; and much more. The Directory includes information on all of the women's foundations worldwide, [and] Regions covered include: Africa, Asia and the Pacific, Europe, North and South America, and the Middle East. Data for this reference work was compiled from details submitted by national and international women's organizations, information gathered from the internet, and directly from individuals holding key positions in major women's organizations* (Research and Markets, 2012, p. 2).

Because not all women's NGOs may have had working websites when this directory was published, we note that the *International Directory of Women's Organizations* likely underestimates the number of women's NGOs worldwide. We compare this with a map of women's NGOs that participated in the 2010 CSW events to see if diverse women's NGOs participate in the CSW (United Nations, a)^6^, showing under-representation of women's NGOs from the UN regions of Latin America, Africa (particularly Northern Africa), and the Eastern European Group (particularly Russia), and the overrepresentation of the United States.

Skeptics may argue that women's NGOs participation at the 2010 CSW session itself is sparse or less diverse because women's NGOs have already exercised their true influence by way of advance regional consultations. It is possible that women's NGOs from a region or country do not attend the CSW because they know other similar groups from their region will attend, and they can get their views advanced during advance consultations. To address this criticism, we developed and fielded an online semi-structured survey interview questionnaire, asking the same survey questions to both women's NGOs who did and did not participate in the 2010 CSW. Ultimately, we were able to get interview responses from 26 attending and 10 non-attending women's NGOs about their views on and participation (or not) in UN activities, as well as their policy priorities for women. To ensure simple, clear English-language questions, we used snowball survey methods to send a draft questionnaire to individuals with a college education who were not born in the United States. In the final version, we asked NGO leaders eight questions, including the home country of the NGO, and semi-structured open-ended questions on the NGO's top three policy priorities, views on how the UN advances women's interests, views on how the UN fails to advance women's interests, and whether or not the NGO had participated in UN events^7^.

We developed this questionnaire drawing on the semi-structured survey method (Rincker, 2017) which combines the virtues of asking respondents the same question for reliability purposes, while allowing for extended open-ended responses to gain unique insights and perspectives on issues that political actors need to the ability to express, as they shape understanding of policy priorities. In interpreting the number of respondents we keep in mind that although from a public opinion point of view, a high percentage response rate is the gold standard, for elite interviews smaller numbers (30–50 interviews) is generally considered high, because the of the quality of in –depth, find-grained information that is gleaned explaining why actors report the outcomes they report.

We fielded the online semi-structured survey interview questionnaire in March-May of 2016, contacting women's NGO leaders in the following way. We carried out this study in accordance with the recommendations of the Purdue University Human Research Protection Program. The Purdue University Institutional Research Board approved the protocol. All subjects gave ***written informed consent*** in accordance with the Declaration of Helsinki. The 2010 CSW listed the names of 445 women's NGOs who participated in the NGO Forum. The CSW provided email address information for 440 of these groups, which we used. Of these, 50 emails bounced back. This left 390 email surveys sent, from which we received 26 unique completed surveys or a response rate of: 6.78%. We examined all 1,760 women's organizations listed in the 2010-IDWO. The Directory listed 723 unique email addresses, but 437 bounced. This left 286 email surveys sent, from which we received 10 unique completed semi structured survey-interviews, a response rate of 3.50%. From a public opinion approach, these response rates are not atypical for online surveys (Deutskens et al., 2004). To try to increase the response rate, we did conduct two follow-up reminders 2 weeks after the initial deadlines to try to increase the number of participants without success. In terms of increasing observable implications (King et al., 1994) on the representativeness and responsiveness of the CSW to diverse women populations, completed questionnaires from 36 women's organizations (26 attending and 10 non-attending) gives us more systematic data on the policy priorities of women's NGOs than researchers have previously collected to the knowledge of the authors.

In order to strengthen confidence in the inferences that we draw with our semi-structured survey interview data, we do two things. We present information showing that our surveys of attending women's NGOs reflect the geographic distribution by UN region of attending women's NGOs, and same for non-attending women's NGOs. The larger disparity we identify is between the regional balance the UN requires for official CSW state delegations (Africa 29%, Asia 24%, Latin America Caribbean 20%, West. Europe Others 18%, East. Europe 9%), and the regional balance in women's NGOs that attended the 2010 CSW (Africa 12.8%, Asia 12.2%, Latin America/Caribbean 4.9%, West. Europe 69 %, East. Europe 1.1%; see Table 1).

**Table 1:** CSW state delegations by UN region and survey response rates by non-attending and attending women's NGOs.

	**Number (%)**					**Total**	**Countries**
	**Africa**	**Asia and the Pacific**	**Latin America and the Caribbean**	**Western European and others group**	**Eastern European**		
State Delegations in the CSW	13 (29)	11 (24)	9 (20)	8 (18)	4 (9)	45	45 countries
Non-Attending Women's NGOs^*^	279 (21.31)	215 (16.43)	63 (4.81)	253 + 418 (51.26)	81 (6.19)	1309	122 countries total; 63 countries which have women's NGO but no attending groups
Non-Attending Survey Respondents	3 (30)	2 (20)	1 (10)	3 (30)	1 (10)	10	10 countries: Uganda, Nigeria, Ghana; Cambodia, India; Mexico; US, Ireland, UK; Georgia
Attending Women's NGOs	57 (12.8)	51 (12.2)	22 (4.9)	162 + 148 USA (69)	5 (1.1)	445	74 countries
Attending Women's NGOs Survey Respondents	2 (7.7)	4 (15.4)	2 (7.7)	18 (69.2)	0 (0)	26	26 countries: Cameroon, Togo; Kazakhstan (2), Pakistan, Thailand; Mexico, Jamaica; USA (9), England, Canada (2), Australia (2), Germany (1), New Zealand (1) Spain (1), Switzerland (1).
Evaluation	Africa underrep	Asia underrep	LA underrep	USA overrep	EE underrep		

*Despite small sample size (26), Surveys of Attending Women's NGOs reflect geographic distribution by UN region of those that attended the 2010 CSW*.

*Surveys of Non-Attending Women's NGOs also reflect geographic distribution by UN region of those that did not attend the 2010 CST. The response rate is low but to be expected since non-attending groups are more numerous that attending groups, and by definition they are less participatory in the UN, so likely less participatory in the survey*.

*The most marked disparity is by State Delegations in the CSW by UN Region and % Women's NGOs Attending the CSW by UN region*.

*US Women NGOs attending the CSW are overrepresented; African, Asian, Latin American, and Eastern European Women's NGO voices are underrepresented. We call for a Fifth World Conference on Women, to increase representation and responsiveness to diverse women populations by UN region, and for the location of that conference to be outside the US and accessible for women of these regions*.

* *Non-Attending Women's NGOs are 2010 IDWO-Attending Women's Orgs minus the attending Women's NGOs*.

Holding the CSW sessions in New York leads to a reality where women's NGOs from the United States are one third of all attending NGOs, and this from a country that has not ratified CEDAW. This regional under-representation of women's NGOs from Africa, Latin America/Caribbean, and Eastern Europe matters. As Bachrach and Baratz (1962) describe, one face of power is the visible face, the decisions that a political body hands down “when A participates in the making of decisions that affect B” (p. 948). The other face of power is the invisible face: the issues that never get on the agenda that affect our lived reality. As Bachrach and Baratz state:

…*power is also exercised when A devotes his energies to creating or reinforcing social and political values and institutional practices that limit the scope of the political process to public consideration of only those issues which are comparatively innocuous to A. To the extent that A succeeds in doing this, B is prevented, for all practical purposes, from bringing to the fore any issues that might in their resolution be seriously detrimental to A's set of preferences (p. 948)*.

In short, control of the agenda is a critical aspect of power. The non-participation of diverse women's NGOs is indicative of policy priorities that do not make it on the CSW's agenda: either through the top-down advance consultations on priority theme, the formal session in New York, or the resolutions passed by the CSW. We show this shortly when we summarize the resolutions passed by the 2010 CSW and compare it with our data on the policy priorities of attending and non-attending women's NGOs.

We also triangulate our survey data with other data that strengthens confidence in the inferences we are drawing. As Tarrow (1995) argues, triangulating different sources of quantitative and qualitative data helps to strengthen confidence in the inferences we could draw on just one type of data. We draw on historical data from the *Short History of the CSW*, which describes the number of NGOs participating in CSW-initiated events over time including the World Conferences on Women held in Mexico City, Copenhagen, Nairobi, and Beijing, which have large attendance by women's NGOs. We contextualize our research with Arat's (2015) analytical table on the breadth of the ideological agenda of the CSW during times of widespread women's NGO participation in CSW policymaking. Reporting on these historical, theoretical and empirical sources in conjunction with the results of our survey of attending and non-attending women's NGOs increases our confidence in drawing inferences that when the number of women's NGOs participating increases, so does the breadth of the CSW's policy agenda. We argue that while valuable, the CSW is most responsive and representative of women when there is a World Conference on Women on the horizon. This institutional design facilitates cross-regional or transnational women's NGO working with the CSW, providing grassroots and transnational feedback on what the priorities or top issues should be. We conclude by arguing for a Fifth World Conference on Women.

## Results

The principal body dedicated to working on gender equality is the CSW, and its principal actors are the 45 country commissioners elected to serve on the Commission. According to Ireland's Gender Equality Machinery website, “[e]very year, representatives of Member States gather at United Nations Headquarters in New York to evaluate progress on gender equality, identify challenges, set global standards, and formulate concrete policies to promote gender equality and women's empowerment worldwide^8^”.

**Figure 1:**
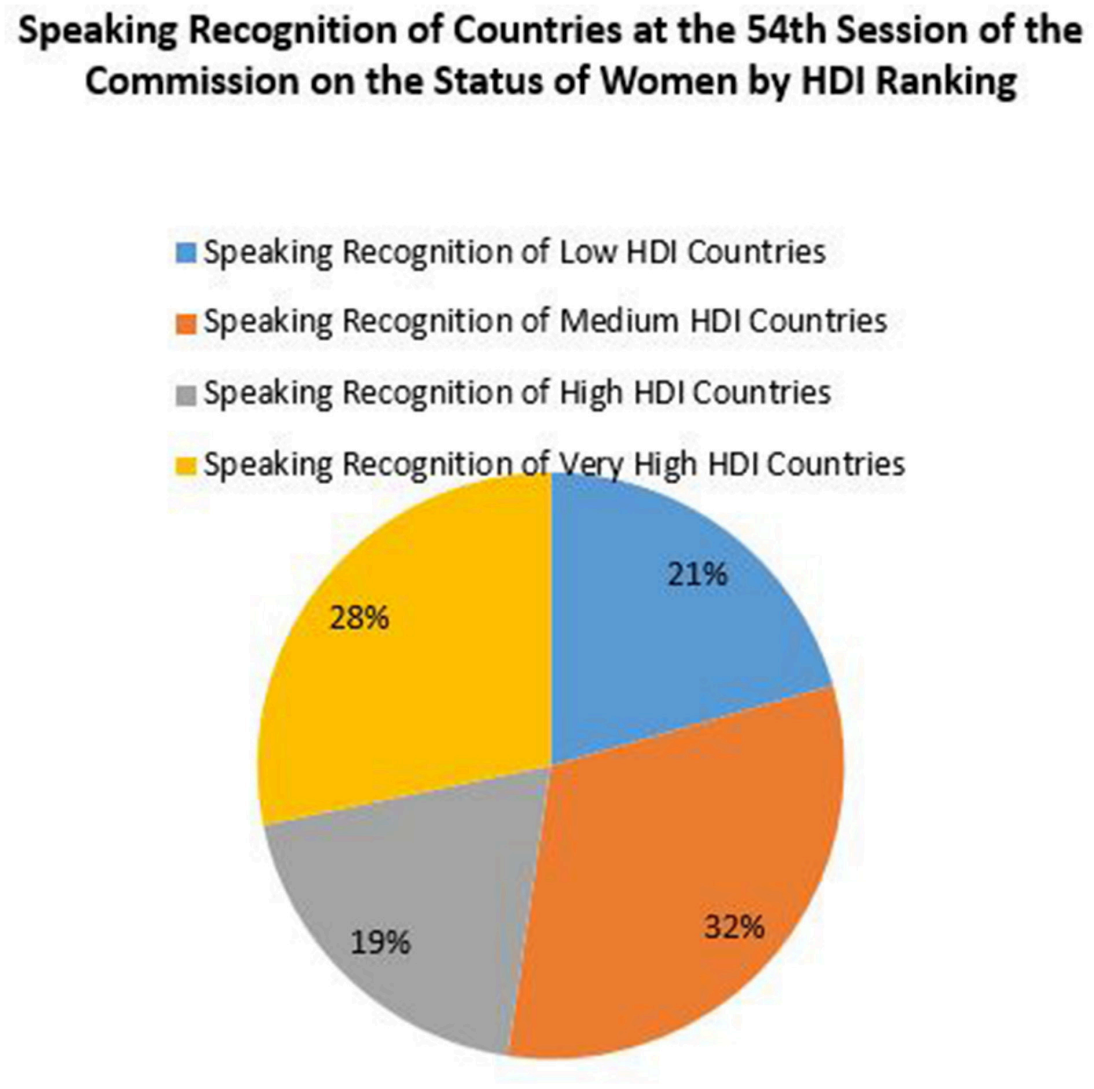
Frequency of speaking time at the 2010 CSW by Human Development Index Rating.

It is possible that states that do not speak that much in session are important to deciding what gets on the agenda, what issues are discussed and how other countries vote. Still, we wanted to examine the CSW as a body to see who speaks and why during CSW formal session. We therefore conducted analysis of speaking time in the formal CSW session to see if there were patterns in which countries spoke. We wanted to see whether wealthier or more economically developed countries dominated speaking time in the CSW, or whether countries controlling the dais called on allying countries in the CSW. Our research indicates that countries with a lower Human Development Index had equivalent speaking time during formal session as countries with higher Human Development Index (see Figure 1).

**Figure 2:**
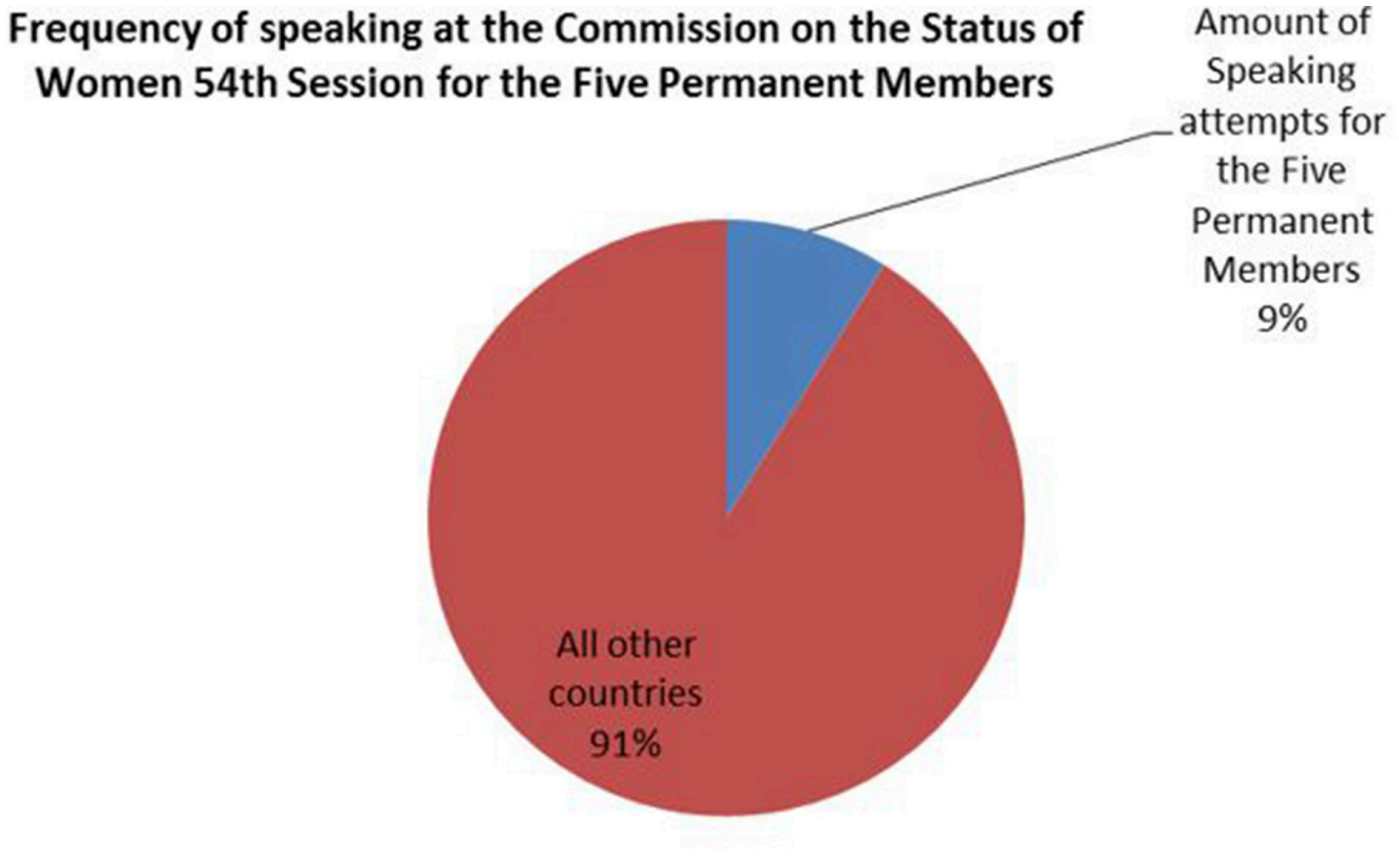
Frequency of speaking at 2010 CSW By P-5 member states.

Furthermore, we find that official state representatives from the Permanent Five member countries (United States, Russia, China, France, and the United Kingdom) spoke 9% of the time in the 2010 CSW session (see Figure 2).

Our analysis therefore shows that during the formal session, the UN CSW allows for the representation of a diversity of women's voices, not just representatives of the P5 countries but non-P5 countries, and this is an important strength of this most important international body working for women's empowerment. As far as the agenda of this formal session, we again note that the Bureau of the CSW is the CSW's leadership, elected for terms of 2 years, and the Bureau meets immediately at the close of each CSW session to elect the new Chairperson and officers, to begin preparations for the following year. According to the ECOSOC Resolution 2015/2016 on the CSW's “method of work,” each CSW session must consist of eight components. The first is a ministerial segment, the second a consideration of one priority theme based on BDPfA and the 23rd special session of the UN General Assembly (GA), and the third is discussion of the 2030 Agenda for Sustainable Development. The fourth component is evaluation of progress in implementing conclusions from previous sessions, the fifth engagement in general discussion of the status of gender equality in light of previous goals, and the sixth, a discussion of new issues affecting women requiring timely response. The seventh component is mainstreaming in the UN System, hearing the Working Group on Communications, agreeing to further actions by adopting conclusions and resolutions, and the eighth is observing International Women's Day if during the formal session (United Nations)^1^,^9^. The design of the brief but formal CSW session encourages high levels of representativeness and responsiveness of diverse women populations as reflected in state delegations' participation in debates. This design reflects norms in the United Nations for regional balance in the CSW's membership. We now move to consider whether the CSW represents and is responsive to diverse women's NGOs during the CSW formal session.

The CSW engages in regional consultations in the lead-up to each yearly March CSW Formal Session. The Committee of the CSW goes to the five regions with the priority theme and data and reports tying that region to the theme. The CSW establishes their priority themes in multi-year programmes of work that are set out far in advance through CSW state deliberations, and not directly in consultation with women's NGOs. On the positive side, the priority theme gives shape and consistency to what the CSW regional consultations and preparatory documents look like, and women's NGOs fit their issues into this theme. On the negative side, a top-down priority theme may limit representation and responsiveness to diverse women's NGOs. First, there may be pressing current issues within a region that do not fit well into the theme and do not get consideration or problem-solving attentions of the CSW and women's NGOs. Second, there may be long-term, slightly lower issues that cross many regions, and in the absence of a space for coordinated transnational women's NGO participation, the UN may perennially ignore these issues just below the radar. While we recognize the CSW for meeting with women's NGOs through regional consultation, we rate the CSW as moderate on responsiveness to women's NGOS in this realm because the CSW determines the priority theme without advance input from diverse women's NGOs.

As of June 2019 the CSW website indicates that both ECOSOC approved women's NGOs and non-approved groups can participate in side events (UN Women, d 2019)^5^ and admittance of non-approved groups is a more recent development. But, generally, the CSW recognizes four possible “modalities of NGO participation” reserved for women's groups that have consultative status granted by ECOSOC (UN Women, e 2019)^10^. The first modality is the aforementioned regional consultations, dates and locations published in the UN Journal. The second modality is submitting an under 2,000 word written statement on the thematic issue 10 weeks prior to the session. The third modality is intervening from the floor during an interactive panel, and this request must be made in advance, the comment should be on the issue theme, and no longer than three minutes, subject to time availability at the CSW. The fourth modality is a request to make a speech during the CSW General Discussion. The statement is limited to no more than three minutes long and should be on the theme topic, preferably on behalf of many NGOs. How has the CSW evolved to a system where, except for a few panels and hand-selected short prepared statements during the CSW Formal Session, on the topic of the CSW's choice, women's NGOs can only listen to state representatives or meet off site, away from actual decision-making?

We acknowledge the CSW, modeling it on UN principles of state sovereignty yet international collaboration, that it is a forum for deliberation to advance state actor's common interests on women's issue. However, we reiterate that *the CSW's own documents argue that it must be responsive to women's NGOs worldwide*. From 1970 to 1994, CSW Commissioners knew that World Conferences on Women were on the horizon, and that parallel transnational activism would be gathering thousands of women's NGOs in advance meetings, the CSW process of advance regional consultation rationally anticipated a broader agenda for diverse women populations. Since 2010, when the CSW has not approved a Fifth World Conference for Women, only hundreds of ECOSOC-approved women's NGOs attend high cost and relatively low impact formal session in New York, at which US women's NGOs constitute a third of the participants, underrepresenting women's NGOs from the regions of Africa, Asia, Latin America/Caribbean, and Eastern Europe. The women's NGOs participate predominantly offsite: at the parallel NGO Forum. In short, the CSW Formal sessions are a place where women's NGOs have an offsite and marginal presence in the process. Our findings echo those of other key scholars who have argued for a Fifth World Conference on Women (Goetz, 2018).

**Figure 3:**
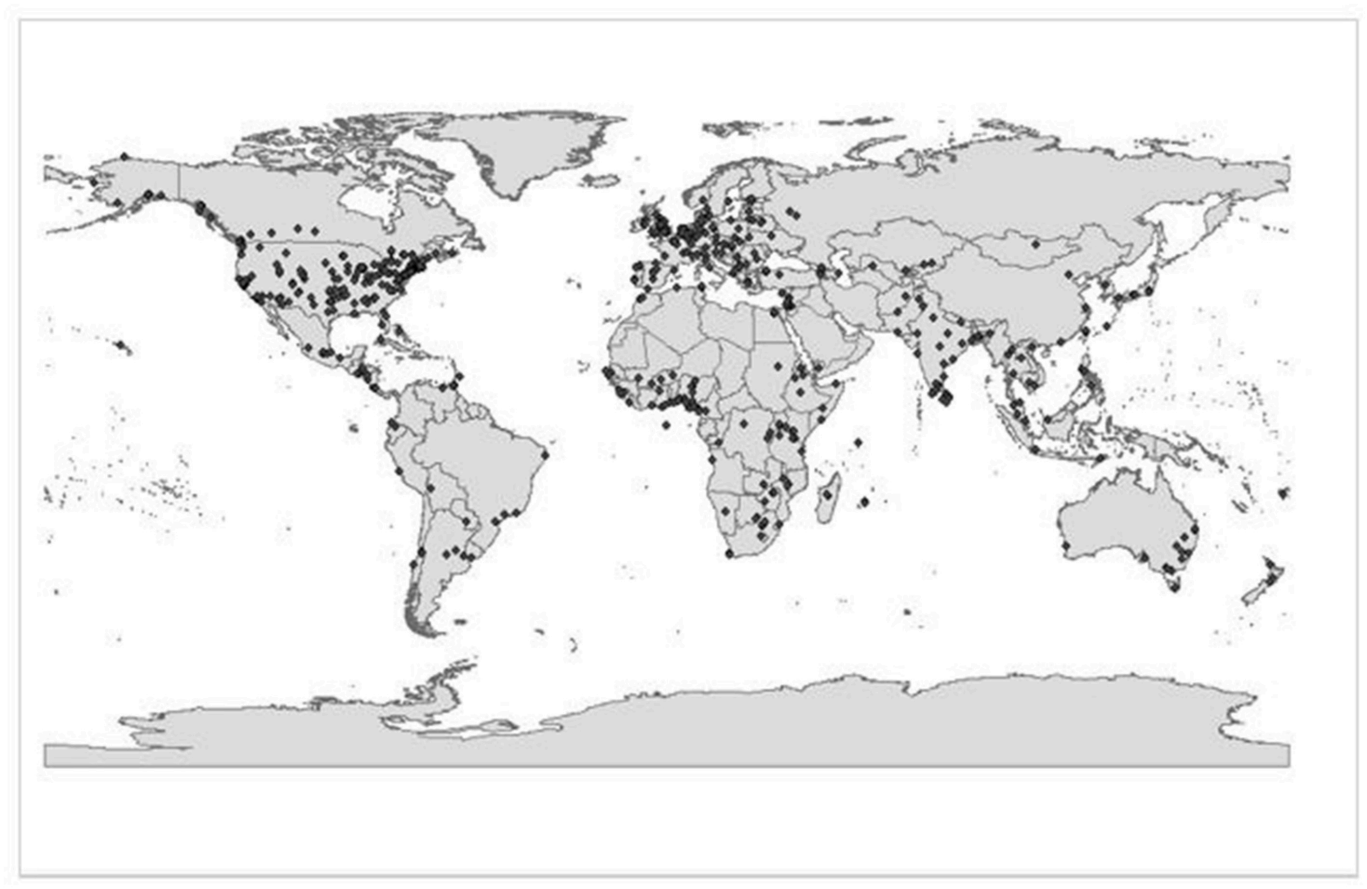
Map of internationally visible women's NGOs, circa 2010. Source: Research and Markets (2012). *N* = 1,760.

If the CSW is responsive to women worldwide, we should observe diverse women's NGOs reflecting regional distributions from UN regions participating in the CSW session, or their policy priorities making it through from advance regional consultations into resolutions at formal session. Using GIS, we identify where internationally visible women's NGOs were located, and compare these with the addresses of women's NGOs that participated in the 2010 CSW NGO Forum. Accordingly, we used ArcView GIS to map data from the 2010-IDWO on the locations of 1,760 internationally visible women's NGOs, which we present in Figure 3.

**Figure 4:**
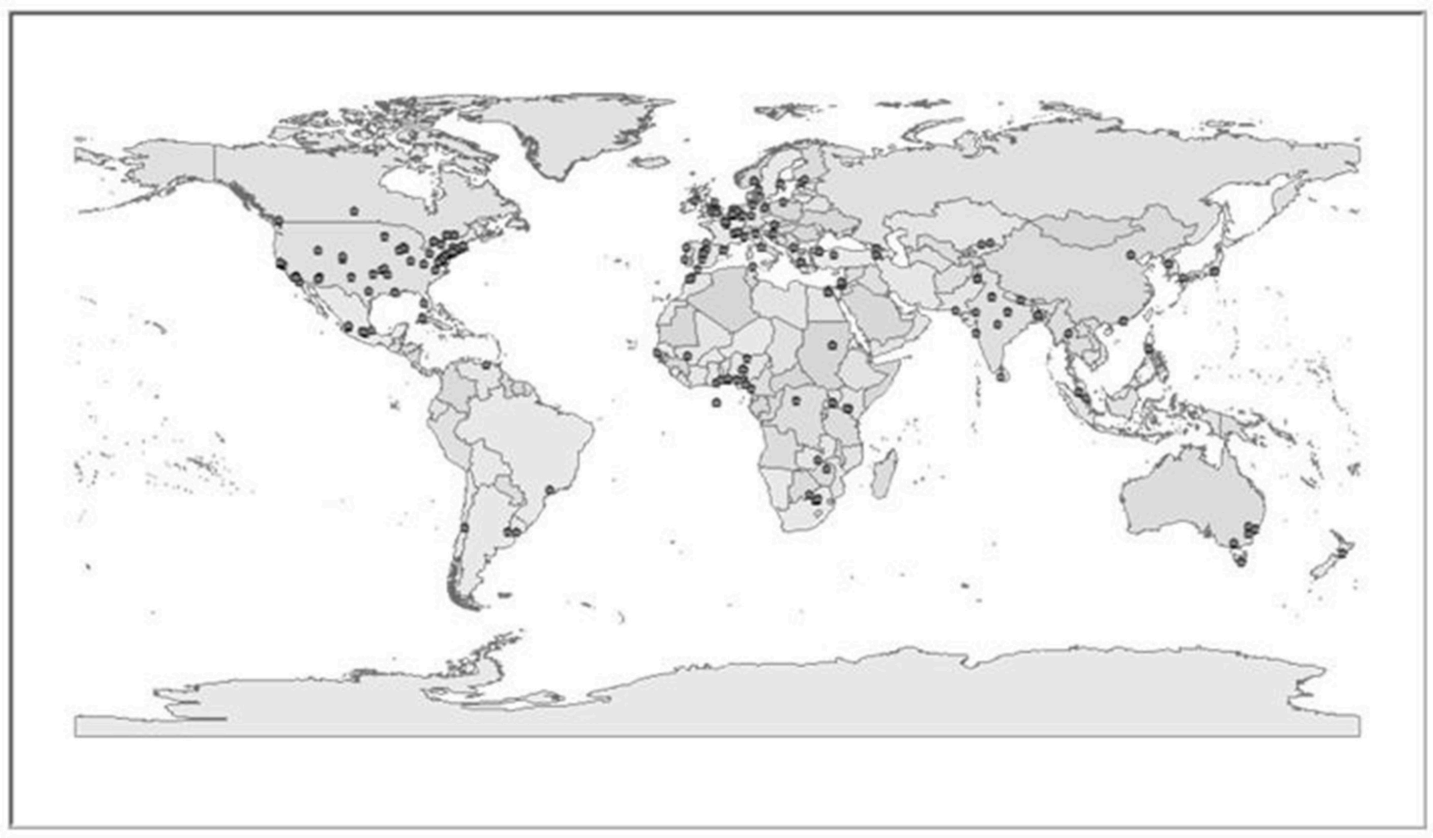
Map of women's NGOs participating in the 2010 CSW NGO Forum. Source: UN Data. *N* = 447.

Using the same process, we mapped the 445 women's groups participating in the 2010 CSW NGO Forum. Examining Figure 4, and comparing it Figure 3, we see that women's NGOs active in the 2010 CSW were mostly from the Western European and others Group (particularly the United States was heavily overrepresented). Technically, the United States is separate from WEOG at times but gathers with this UN regional group. African women's NGOs were under-represented (particularly North Africa). Asian women's NGOs were under-represented (particularly Northern Asia). Latin America was under-represented (particularly outside of Mexico). Women's NGOs from the Eastern European Group (particularly Russia was absent).

What explains the lack of women's NGOs and the lack of diverse women's NGOs by region? About one third (151 or 33.4%) of women's NGOs at the 2010 CSW were from the United States, which makes sense given that travel was least expensive for these groups. The United Kingdom was second with 29 organizations (6.4%). France and China followed with a total of 12 (2.7%) and three groups (0.7%), respectively. Even though there were women's NGOs in Russia, none attended the NGO Forum. Media Rights Voices (2017) argues that UN NGO Committee members including Russia “actively use their Committee membership to stymie applications for UN status from NGOs active in the field of human rights. Using procedural ruses, members of the Committee can, and often do, defer applications session after session with inappropriate, specious or repetitive questions and demands of the NGOs.”

To attend the 2010 CSW, and be considered by the Committee on NGOs at one of their two meetings in 2009, a women's NGO had to apply for ECOSOC consultative status by 1 June 2008. Doing so involves submitting a complete application in English or French including: an online organizational profile questionnaire, a copy of the organization's bylaws, a certificate of registration attesting that the group has been in existence for at least 2 years, and a copy of the NGO's financial statements (United Nations, b)^11^. The Committee may ask the NGO follow-up questions, and the committee must provide notice of organizations it is nominating for consultative status in advance to the ECOSOC, which the ECOSOC ultimately has the power to approve. Although the UN has six official languages: Arabic, English, French, Chinese, Russian, and Spanish, ECOSOC only accepts English or French language applications from women's NGOs seeking consultative status. NGOs need to have existed more than 2 years, produce financial statements and bylaws, and possess predictable electrical and internet access. We do not know which groups applied and were denied so cannot say definitively, but point out that the UN's inconsistent language requirements may inhibit women's NGOs from Spanish, Russian, and Arabic –speaking countries from participating in the most important international body working for women's empowerment.

After ECOSOC approval, travel costs to attend the CSW's meetings can be burdensome. From our survey, half of organizations that did not attend the 2010 CSW indicated lack of funding as the main reason. Seven of the 26 attending organizations indicated funding was the main barrier preventing wider participation in UN events. As noted by the Bangkok based Women Organizing for Change in Agriculture and Natural Resource Management (WOCAN), “[…] I do not perceive the UN as a body that is so open to ideas. Traveling to the CSW [New York] is very expensive and difficult for many to get US visas. [Moreover,] other events around the world are not frequent.” If CSW held its meetings in a variety of locations, like the World Conferences on Women, participation by women's NGOs may expand.

The United Nations has taken on a primary role in development of treaties and agreements to promote the progress of humanity on a global scale. During its 71 years, the CSW has changed from its 1,946 origins in which “the first meeting was open to the public, with 250 seats open to the general public and 250 seats reserved for members of “educational groups and voluntary accredited organizations” (qtd. in Baldez, 2014, p. 48) to one in which women's NGOs must apply to ECOSOC for consultative status. From 1975 to 1995, over the course of four World Conferences on Women, women's NGO participation grew from 6,000 NGO representatives in Mexico City, to 8,000 in Copenhagen, 12,000 in Nairobi, and 30,000 in Beijing. However, as of 2017, periodic CSW-led reviews of the Fourth World Conference, and sporadic regional consultations between UN Women and women's NGOs preempt direct, broad participation of women's NGOs at the CSW.

As a result of moving outside of some of the external and internal obstacles that the CSW faces, the World Conferences on Women allowed for the CSW to become more responsive and representative to diverse women populations. As Table 2 shows, starting with the First World Conference on Women in Mexico City (1975) the rhetorical agenda for women's rights grew much broader. The rhetorical agenda moved from a liberal feminist focus on women's rights, to Marxist, socialist, and post-colonial feminist approaches that emphasized the need for women in development, women's role in preventing and solving conflict, and the effects of race and class on women. When women's NGO presence grew at World Conferences on Women, the CSW's power in the UN system adjusted accordingly. “From 1970 to 1986, the [CSW] only met biannually” as it was not coordinating the World Conferences for Women, and “some states even proposed the abolition of the Commission in 1980, and argued for transferring its functions to ECOSOC.” However, at the 1980 World Conference on Women in Copenhagen, “they recommended the Commission be strengthened and given full responsibility for the preparation of [the 3rd World Conference on Women in Nairobi]” (United Nations, b, p. 11)^3^. Women's NGO participation numbered 30,000 in Beijing in 1995 (UN Women, f)^12^, and the UN gender equality agenda expanded. The BDPfA outlined 12 areas of concern, reflecting a broad agenda, including not just traditional liberal feminist areas like women in power and decision-making, education and training of women, institutional mechanisms for the advancement of women, women and health, women and the media. Additionally, the BDPfA highlighted areas of higher concern to socialist, postcolonial, and intersectional feminisms, such as: women and the environment, the girl child, women and the economy, human rights of women, violence against women, women and armed conflict, and women and poverty (UN Women, f)^12^.

**Table 2:** NGO participation, Feminist approach, and Breadth of UN Gender-Equality Policy Agenda, 1946–2015.

**Time period**	**NGO participation rules and numbers of NGOs in CSW events**	**Feminist approach**	**CSW-led UN gender equality agenda**
1947	250 seats open for NGOs, several reps. attend	Liberal feminism	• Participate in drafts of Universal Declaration of Human Rights
1948–1959	ECOSOC consultative status required; 30–50 NGO reps. attend		• Convention on Equal Remuneration (1951) • Convention on the Political Rights of Women (1952) • Convention on the Nationality of Married Women (1957)
1960–1969	ECOSOC consultative status; required 30–50 NGO reps. attend	Marxist Feminism	• CSW works with UNESCO on women's literacy, education • Women's economic participation and poverty • Research assessing status of women worldwide • Convention on Consent to Marriage, Minimum Age for Marriage and Registration of Marriages (1965) • Declaration on the Elimination of Discrimination against Women (1967)
1970–1974	ECOSOC consultative status; 30–50 NGOs reps. attend	Radical Feminism	• CSW considers Bose's *Women's Role in Economic Development* (1970)
1975–1979	6,000 NGO reps at International Women's Year Tribune	Socialist Feminism	• First World Conference on Women, Mexico City (1975) • UN Decade for Women:Equality, Development, Peace • CSW working groups draft CEDAW with the GA Third. • *CEDAW* (1979)
1980–1984	8,000 NGO reps. attend Copenhagen NGO Forum	“Women of Color” Feminisms	• Second World Conference on Women, Copenhagen (1980) focus on employment, health, education • Programme of Action: women's ownership, inheritance, child custody, loss of nationality
1985–1989	12,000 NGO reps. attend Nairobi NGO Forum	Third World/Post-Colonial Feminisms	• Third World Conference on Women, Nairobi • Nairobi Forward-looking strategies, mainstreaming women's equality, exposing violence against women
1990		“Women of Color” Feminisms	• Declaration on the Elimination of Violence Against Women. Mainstreaming at development/environmental conferences.
1995	30,000 NGO reps.attend Beijing NGO Forum	Third World /Post-Colonial Feminisms	• Fourth World Conference on Women: Beijing Declaration and Platform for Action's 12 critical areas of concern. • Optional Protocol to CEDAW (1999)
2000	2,000 NGO reps. attend	Intersectionality	• 23rd special session of GA • Security Council Resolution 1325, Millennium Development Goals
2005			Beijing +10
2010	479 NGO reps. attend		Beijing +15
2015	477 NGO reps. attend		Bejing +20 UN Women, People's Republic of China organize state Commitment to Action

*Sources: Feminist Approach printed previously in Arat (2015); United Nations (b)^2^; UN Women (a)^1^; UN Women (f)^12^. Section of Arat (2015) is being used with the permission of the copyright holder Cambridge University Press. Others wishing to cite should seek separate permission clearance*.

Since halting the practice of World Conferences on Women, CSW-led, state-centric reviews of Beijing have limited the ability of women's NGOs to work with the CSW to craft policy. Since 2001, the number of women's NGOs attending CSW sessions has remained low: from 136 groups in 2002 to 479 in 2005 (United Nations, a)^6^. In 2015, the CSW organized a 20 year review of Beijing, but more attention was focused on an event jointly sponsored by UN Women and the People's Republic of China Gender Equality and Women's Empowerment: A Commitment to Action. This process was led by UN Women and state leaders, and sidelining women's NGOs further. When the CSW increases its representation and responsiveness to diverse women's populations through women's NGOs, it does its work more effectively.

In this section, we examine whether the seven resolutions passed by the 2010 CSW responded to the top priorities of women's NGOs. We report from original surveys the top policy priorities (ranked 1–5 by frequency) of women's NGOs in two groups, those that attended (a) and those that did not attend (na) the 2010 CSW. Items with an asterisk indicate a priority not shared by the other group. For attending groups (a): Priority 1 was eliminating violence, sexual assault, and spousal abuse. Priority 2 was women's health. Priority 3 was education^*^. Priority 4 was political representation and land rights. Priority 5 was maternity leave and wages. For non-attending groups (na): Priority 1 was eliminating violence, sexual assault, and spousal abuse. Priority 2 was political representation and land rights. Priority 3 was maternity leave and wage equality. Priority 4 was women's health. Priority 5 was economic development and poverty^*^. There was a great deal of overlap in top priorities of women's NGOs. A responsive CSW should have a particular urgency to address these shared priorities: *eliminating violence against women, political representation and land rights, women's health, and maternity leave and wage equality*.

The 2010 CSW adopted the following seven resolutions (Commission on the Status of Women, 2010).

1. CSW Resolution 54/1. Declaration on the occasion of the fifteenth anniversary of the Fourth World Conference on Women
  This resolution reaffirmed the importance of the BDPfA on the occasion of its 15th anniversary, noting that more progress was needed.
2. CSW Resolution 54/2—Women, the girl child and HIV and AIDS
  This resolution recognizes the disproportionate vulnerability of women and girls to the HIV and AIDS pandemic worldwide, as both a cause and a consequence of poverty and other social and economic disadvantages. The resolution calls for governments and civil society to intensify efforts and international cooperation aiming to promote universal access to comprehensive HIV prevention programs and treatment, as well as political and financial coordination to address gender equality in national HIV and AIDS policies.
3. CSW Resolution 54/3. Release of women and children taken hostage, including those subsequently imprisoned, in armed conflicts
  This resolution condemns hostage taking, requests states take all possible measures to rescue hostages, and to collect gender and age disaggregated data on hostage victims.
4. CSW Resolution 54/4—Women's economic empowerment
  This resolution acknowledges that the empowerment of women is a critical factor in the eradication of poverty. It urges member states to ensure women and girls equal access to education, basic services, including primary health care, housing, economic opportunities and decision-making at all levels, requesting states to adopt and apply effective measures to ensure the application of the principle of equal remuneration for men and women workers for work of equal value.
5. CSW Resolution 54/5—Eliminating maternal mortality and morbidity through the empowerment of women
  This resolution recognizes that half a million women and adolescent girls die every year from preventable complications related to pregnancy or childbirth. Over 200 million women lack access to safe and effective forms of contraception. It stresses the need to combat discrimination, poverty, and harmful traditional practices and acknowledges the critical role of men and boys in supporting women's access to safe conditions for pregnancy and childbirth.
6. CSW Resolution 54/6 Strengthening the institutional arrangements of the United Nations for support of gender equality and the empowerment of women by consolidating the four existing offices into a composite entity.
  This resolution reaffirmed CSW support for the creation of UN Women, a focal agency for research, advocacy and gender mainstreaming in the UN system.
7. CSW Resolution 54/7—Ending female genital mutilation
  This resolution identifies female genital mutilation as a violation of girls' and women's bodies, causing irreparable harm to an estimated 100 million women around the world. This resolution calls for states to take all necessary measures to enact and enforce legislation to prohibit FGM and to protect girls from the practice.

We find that the CSW adopted resolutions on *some but not all priorities shared by attending and non-attending women's groups*. The CSW adopted resolutions in the areas of Priority 1 on eliminating violence against women (Resolution on Female Genital Mutilation), Priority 2 on women's health (Resolutions on HIV/Aids and Resolution on Maternal Mortality), and Priorities 3 and 4 on maternity leave and wages; as well as emphases on education and poverty (Resolution on Economic Empowerment). However, the CSW did not address the second highest priority of women's NGOs: that of land rights. We argue that the CSW's state-centric orientation means that it does not take substantial action on women's priorities if they contravene traditional territorial rights within states.

While recognizing the 2010 CSW's work on resolutions, we note two objections. First, in some cases a resolution is incomplete: addressing one aspect of an issue identified by women's NGOs as without additional resolutions. The resolution on FGM was important, but the 2010 CSW did not pass additional resolutions on sexual assault and spousal abuse outside of the context of FGM itself, even though both attending and non-attending groups reported that their Priority 1 was eliminating violence, sexual assault and spousal abuse. Second, the bundling of three broad policy areas into one omnibus resolution gives the individual issues short shrift, and limits the ability of women's NGOs to challenge their home state's records on women and girls in specific policy areas. While the Economic Empowerment resolution briefly mentions the need for women's equal access to equal wages, education, and poverty all within one resolution, it does not go into any specifics as to why these inequalities exist and what specific policies would remedy them. By bundling these broad policy issues, the CSW and women's NGOs lose the ability to call out their home governments. States can point to any initiative that “economically empowers women” as fulfilling the resolution without changing underlying inequalities.

The CSW did not pass resolutions on key priorities articulated by women's NGOs, which we argue reflects the second face of power, or issues that are not getting on the CSW's agenda in its current institutional format, especially because of non-attending women's NGOs. First, the CSW did not pass any resolutions dealing with land rights, Priority 4 of attending women's groups but Priority 2 of non-attending women's groups. Such a resolution would require a fundamental transformation of patriarchal institution of the state, which tends to equate power with land, and to transfer land and property from elderly males to younger first-born males. Reforming inheritance and land rights in some developing nations could lead to major internal conflicts within and between states. This reality reflects Arat's discussion that “while many Third World/transnational feminist concerns are articulated in UN outcomes documents, these documents have been silent on class oppression or fail to mention capitalism by name as a contributor to the hardship faced by many women” (p. 686). Because of our focus on the second face of power, our empirical evidence shows that despite support across both attending and non-attending groups, a resolution on land rights did not get on the agenda for internal and external reasons. Internally, countries wouldn't support women's groups to attend the CSW with this as a top policy priority, but also externally, such a resolution passed with the CSW contravenes the CSW's and UN's focus on the international system being one of sovereign states and involves transformational change: post-colonial feminism rather than liberal feminism.

The CSW also did not pass resolutions on other issues listed by non-attending women's NGOs: the trafficking of women and girls, internet access for women around the world, and the effects of climate change on women and girls. These policy priorities also deal with flow of resources across traditional state boundaries. Perhaps political actors will take up these issues through gender mainstreaming efforts on Paris Talks on Climate Change or conventions on the digital divide. However, as the most important international body charged with women's empowerment, a responsive CSW should pass resolutions on these top priority issues of trafficking women and girls, internet access for women and girls, and the gendered effects of climate change.

Our surveys showed that women's NGOs believed that the CSW reflected the priorities of liberal women's NGOs, rather than inviting diverse critical women's NGOs:

[i]nfluence at the UN is hard won. Good ideas do not rise to the top. Long-term efforts, particularly with delegates, individuals in their home capitals, grassroots coalitions, and work inside the UN itself is needed to try to influence. Even then, significant influence is provided by powerful, well-funded international NGOs who dominate the questions related to women's health, rather than the women to whom programs are normally targeted (developing nations). The UN operates much as other political spheres and it is difficult to achieve evidence-based success in the decisions that are taken.

Another women's NGO stated: “[t]he United Nations does not appear to address worldviews, theologies or beliefs that support violence and patriarchal attitudes toward women, and promote political violence.” Yet another women's NGO leader stated that their top priorities were harassment by husbands, sexual violence in the workplace, and forced entrance into the sex trade, issues not tackled head-on by the 2010 CSW. A women's NGO from a marginalized and poor region of a developing country stated that women bear the brunt of conflict, and have influence in preventing conflict, but are not included in UN discussions^13^. This group wanted to attend the CSW but could not afford it and “had never been invited.” Statements by attending and non-attending women's NGOs underline our assessment that the CSW privileges state actors for women and NGOs supportive of liberal feminism.

Initially on the periphery of policy-making, World Conferences on Women grew out of a state delegation responding to a request from a well-organized women's NGO federation with offices around the globe. At the CSW's first meeting, a federation of women's organizations was present. This group was the Women's International Democratic Federation (WIDF): a federation with member organizations from 70 plus countries and executive board memberships from all regions, including Western Europe. The WIDF was not allowed to participate in subsequent CSW sessions because it was deemed a “communist front,” (Baldez, 2014, p. 49) so the Romanian state delegation on behalf of WIDF proposed that the UN General Assembly (GA) designate 1975 International Women's Year to remind the world of persistent gender inequalities (United Nations, c, p. 88). This idea was not only endorsed by the CSW, but grew into a GA-endorsed First World Conference on Women in Mexico City (1975), a UN Decade for Women (1976–1985), punctuated by the Second World Conference on Women in Copenhagen (1980), and culminating in the Third World Conference on Women in Nairobi (1985). Basu argues the “most productive global influence on women's movements have been United Nations' support for women's rights and women's conferences” (Basu, 2010, p. xxi) At the World Conferences on Women, women's NGOs from all over had a chance to meet, network, and strategize (Lycklama i Nijeholt et al., 1998; see Keck and Sikkink, 1999). During the consultative process in the lead-up to the 1995 Beijing Conference, women's NGOs met in regional groupings to discuss their priorities and knowledge of UN processes, coalitions, and allies. During this time that women's movement actors drafted, prepared and negotiated the outcome document of the conference, the Beijing Declaration and Platform for Action (BDPfA).

Throughout the UN gender equality policy-making process, state-selected representatives on women's rights in the GA Third, CEDAW, and UN Women directly interface with one another to influence gender equality policy at multiple levels. Women's NGOs with significant resources may be in a position to work with or influence the GA, CEDAW, and UN Women if the women's organizations themselves are large and well-financed to get the attention of their state delegates to the UN, or through consultative processes as members of the GA draft numerous resolutions dealing with women's rights. Because in the UN, state representatives on women's issues subordinated women's NGOs, the UN ultimately upholds the power of states. Diverse women's NGOs such as those that challenge the state as patriarchal do not have the opportunity to meet with CSW commissioners. The ECOSOC process for granting consultative status filters such women's NGOs out of the process of representation.

## Recommendations

In this article, we use a mixed-method approach to evaluate the level of United Nations Commission on the Status of Women (CSW) representation and responsiveness to diverse women populations as reflected in (a) state delegations' participation in debates, (b) NGO participation, and (c) NGO policy priorities. We find that the CSW is highly representative and responsive to diverse women populations in terms of state delegation participation across UN regions. We find that the CSW is at best moderately representative and responsive to women's NGOs. On one hand, the CSW conducts regional advance consultations, but on the other women's NGOs face costly barriers to entry to gain ECOSOC status, attending women's NGOs are not diverse across UN regions, and those that do participate are primarily involved in parallel off-site events during CSW formal session. Although the 2010 CSW passed important resolutions in some areas, we find its level of representation, and responsiveness to women's NGO policy priorities is low. We base this evaluation on our surveys of attending and non-attending women's NGOs that show top policy priorities the 2010 CSW did not discuss or pass resolutions on including land rights, sex trafficking, internet access for women, and the effects of climate change on women. We contend that during 1970–1994, when World Conferences on Women occurred, parallel transnational advance consultations among women's NGOs in tandem with CSW regional consultations created a broadening of the CSW's agenda. The CSW rationally anticipated a broader set of priority issues for diverse women populations. Further research to explore UN gender equality policymaking is needed, but in the meantime, we argue for a Fifth World Conference on Women, so that CSW work can represent and respond to cross-regional women's NGO activism and solutions.

## Ethics Statement

This study was carried out in accordance with the recommendations of the Purdue University Human Research Protection Program with written informed consent from all subjects. All subjects gave written informed consent in accordance with the Declaration of Helsinki. The protocol was approved by the Purdue University Institutional Research Board.

## Author Contributions

MR, MH, RV, and DD all contributed to the conception and design of this study, the GIS data collection and analysis, online survey questionnaire and analysis, and writing sections of the manuscript. All authors contributed to manuscript revision, read and approved the submitted version.

### Conflict of Interest Statement

The authors declare that the research was conducted in the absence of any commercial or financial relationships that could be construed as a potential conflict of interest.

## Footnotes

^1^UN Women (a). *Commission on the Status of Women*. Available online at: http://www.unwomen.org/en/csw (accessed June 15, 2019).

^2^To date, 187 out of 193 countries have ratified or acceded to CEDAW [Feminist Majority. CEDAW (The Women's Treaty). Available online at: https://feministmajority.org/our-work/cedaw-the-womens-treaty/ (accessed June 15, 2019)]). The CEDAW does not discuss violence against women as topic, but the Preamble does condemn the discrimination of women as it violates the principles of equality of rights and respect for human dignity.

^3^United Nations (b). *Short History of the Commission on the Status of Women*. Available online at https://www.un.org/womenwatch/daw/CSW60YRS/CSWbriefhistory.pdf

^4^Un Women (c). 2019. *Member States*. Available online at: http://www.unwomen.org/en/csw/member (accessed June 15, 2019).

^5^UN Women (d). 2019. *NGO Participation*. Available at: http://www.unwomen.org/en/csw/ngo-participation (accessed June 15, 2019).

^6^United Nations (a). *Advancement of Women*. Available online at: http://esango.un.org/civilsociety/displayAowSearch.do?method=search&sessionCheck=false

^7^No groups selected our option to have survey questions translated and to respond in Spanish. Time and budgetary constraints prevented us surveying in all six official UN languages.

^8^Gender Equality in Ireland (2019). Commission on the Status of Women. Available online at: http://www.genderequality.ie/en/GE/Pages/CSW (accessed June 19, 2019).

^9^Although the CSW has repeatedly sought from the Human Rights Council (before 2006 known as the Commission on Human Rights) the ability to hold individual states accountable, the CSW does not have the authority to do this. The CSW sends its resolutions and reports on to ECOSOC for follow up and consideration by the General Assembly.

^10^UN Women (e) (2019). *Opportunities for NGOs to Address the Commission*. Available online at: http://www.unwomen.org/en/csw/ngo-participation/written-and-oral-statements (accessed June 15, 2019).

^11^United Nations (b). *How to Apply for Consultative Status*. Available online at: http://csonet.org/?menu=83

^12^UN Women (f). *The Beijing Platform for Action: Inspiration for Then and Now*. Available online at: http://beijing20.unwomen.org/en/about (accessed June 15, 2019).

^13^On 31 October 2000, the UN adopted Security Council Resolution 1325 (S/RES/1325), recognizing that gender–based violence is a tool of war and conflict that must be eliminated and addressed by the participation of women at all levels of political decision-making, in militaries, and in peace-building processes.
